# A Cross-Sectional Study Assessing Antibiotic Resistance Awareness Among University Students in Samborondón, Greater Guayaquil, Ecuador

**DOI:** 10.3390/antibiotics14050440

**Published:** 2025-04-27

**Authors:** Norka Michelle Mora Pincay, José Luis Villegas, César Marcelo Larrea-Álvarez, Daniela Beatriz Briones Caiminagua, Lilibeth Torres-Elizalde, Miroslava Anna Šefcová, Marco Larrea-Álvarez

**Affiliations:** 1 Facultad de Ciencias de la Salud, Carrera de Medicina, Universidad Espíritu Santo, Samborondón 092301, Ecuador; 2 Institute for Physical and Information Technologies, Spanish National Research Council, 28006 Madrid, Spain; 3 Laboratoire de Microbiologie et Génétique Moléculaires (LMGM), UMR5100, Centre de Biologie Intégrative (CBI), Université de Toulouse, Centre Nationale de la Recherche Scientifique (CNRS), 31400 Toulouse, France

**Keywords:** antibiotic resistance awareness, health literacy, students, cross-sectional survey, KAP, Ecuador

## Abstract

**Background/Objectives:** Education on antibiotic use has the potential to positively shape the practices and perspectives of future professionals. Assessing awareness levels of antibiotic resistance among university students is, therefore, critical, as they represent a vital demographic capable of influencing public health outcomes, especially in low- and middle-income countries. **Methods:** This cross-sectional study employed the World Health Organization’s Antibiotic Resistance: Multi-Country Public Awareness Survey, which examines demographics, antibiotic use, knowledge, perspectives, and sources of information. A total of 922 surveys were collected from students across various disciplines at two universities in Greater Guayaquil. **Results:** Most participants reported obtaining antibiotics through healthcare professionals, adhering to proper usage instructions, and purchasing them primarily from pharmacies. However, only 56% of the responses were correct, with many students incorrectly associating antibiotic use with conditions where they are typically ineffective. Despite these gaps, the students expressed positive attitudes toward proposed measures to address antibiotic resistance. While the participants demonstrated familiarity with terms related to antibiotic resistance and identified doctors and educators as their main sources of information, educational campaigns were not widely recognized as important. **Conclusions:** These findings evidence knowledge gaps among an essential group, suggesting the need for targeted health programs, preventive strategies, and educational initiatives to combat misinformation regarding antimicrobial resistance.

## 1. Introduction

The emergence of resistance to common antimicrobial drugs presents a major obstacle to public health. Accordingly, one of the primary goals of the World Health Organization (WHO) is to contain its spread. As a result, antimicrobial resistance (AMR) has been recognized as a critical problem [1,2]. Exposure to antimicrobial compounds drives the selection of resistant microorganisms. In bacteria, resistance genes can emerge through random mutations in the genome or be transferred within and between DNA molecules via mobile genetic elements such as transposons and insertion sequences [3,4]. Furthermore, these resistance traits can spread within bacterial populations through horizontal gene transfer, involving integrative conjugative elements and plasmids [5]. It has been suggested that major contributors to the global dissemination and expansion of AMR include the misuse and overuse of antibiotics [6,7].

Primarily due to the high prevalence of infections, widespread antibiotic availability, a lack of standardized prescribing guidelines, and restricted access to affordable diagnostic tests, public health challenges will be most significant in low- and middle-income countries [8,9]. Addressing AMR in resource-limited settings is essential, as it poses a significant risk of mortality [10]. Ecuador, in particular, has implemented national policies and action plans aimed at controlling the spread of antibiotic resistance. Furthermore, the country’s health regulations guarantee the responsible use of medications by mandating that they be sold exclusively through authorized vendors. The law also requires prescriptions from licensed healthcare professionals for most medications, with the exception of those classified as over-the-counter medications [11,12,13]. Despite these regulations, improper antibiotic use has been documented in various populations across Ecuador [14,15].

It has been reported that education on the proper use of antibiotics can positively influence the perspectives and practices of future professionals regarding antibiotic resistance among university students [16], while biased attitudes and perspectives may also affect antibiotic usage [17]. As a result, numerous studies have aimed to assess the knowledge, attitudes, and practices (KAPs) related to antibiotic resistance among undergraduate students, with the majority concentrating on students in medicine and pharmacy programs [18,19,20,21,22]. These studies have focused on identifying critical issues, thereby providing valuable insights for the development of future health programs and intervention measures. Effectively sharing crucial information, especially regarding the relationship between antibiotic misuse and the emergence of AMR, will be essential for optimizing these efforts [23,24].

Tackling antibiotic resistance demands interdisciplinary collaboration. Law students can contribute to shaping and enforcing regulations to prevent antibiotic misuse, while journalism students can develop effective public communication strategies about the associated risks. Such cross-field cooperation follows the WHO’s One Health strategy, which connects human, veterinary, and environmental health actions [1]. In Ecuador, a moderate level of knowledge has been determined among medical students [25,26]. Similar responses, as well as positive perspectives, have been reported among a general undergraduate population in Quito. However, it was determined that students in applied sciences scored higher than their counterparts in social sciences [27], a trend also observed among Ecuadorian students regarding inheritance and SARS-CoV-2 [28,29]. A comparable disparity in knowledge of AMR has been noted between biology students and those from non-biology disciplines in other countries [18,30]. Antibiotic resistance is a critical global health challenge; however, limited data exist on the knowledge, attitudes, and perspectives of undergraduate students in low- and middle-income countries like Ecuador, particularly in Greater Guayaquil. Assessing the current awareness levels among these students is crucial, as they represent future professionals who can influence public health outcomes. By analyzing a multidisciplinary student population, results could be obtained that offer unique insights, informing the design of early educational interventions to address common misconceptions and mitigate the spread of antibiotic resistance.

In this study, we used the WHO’s “Antibiotic Resistance: Multi-Country Public Awareness” questionnaire, previously applied among undergraduates [27,31,32], to assess knowledge, use, information sources, and attitudes regarding antibiotics in a general population of university students from two institutions in Samborondón, Greater Guayaquil.

## 2. Results

### 2.1. Demographics

A total of 922 surveys were collected (File S1, Supplementary Results, Answers). The largest group consisted of individuals aged 25 and older, followed by those between 19 and 21 years. This could be associated not only with older students being more likely to complete the email-based survey, but also with the inclusion of postgraduate students in our sampling. Students younger than 18 and those aged 22 to 24 made up similar proportions of the population. Females accounted for 65% of all the interviewees, while 61% were enrolled in social sciences programs (Table 1).

### 2.2. Antibiotic Use

Antibiotic use in the past month was reported by 37% (n = 341) of the participants, while 34% (n = 312) indicated usage within the previous six months, and 11% (n = 102) within the past year (Q1). A total of 638 participants (70%) stated that they obtained antibiotics from a healthcare professional, such as a doctor or nurse, while the remaining either could not remember (6%, n = 55) or acquired them through other means (24%, n = 219) (Q2). However, 80% (n = 732) of the respondents confirmed receiving guidance from a general practitioner, whereas 5% (n = 46) could not recall, and the remaining 15% (n = 137) reported not receiving any advice on antibiotic use (Q3). Most students (89%, n = 823) stated that they obtained antibiotics from a medical store or pharmacy, while a smaller proportion had them stored at home (4%, n = 40) or received them from a friend or relative (3%, n = 26) (Q4) (File S2, Supplementary Results, Data).

### 2.3. Sources of Information

Eighty-seven percent (n = 800) of the population were familiar with terms such as antibiotic resistance, antibiotic-resistant bacteria, superbugs, AMR, and drug resistance. However, most participants could not recall where they had heard these terms (Q9). Doctors and teachers were commonly mentioned, while specific campaigns were among the least popular sources (Q10) (File S2, Supplementary Results, Data).

### 2.4. Knowledge of Antibiotics and Resistance

The overall correct response rate was 56%, with a median score of 8.40 (4.8) out of 15 questions. Significant differences were observed by sex, age, and field of study. The male students averaged approximately nine points compared to the females’ eight points. The healthcare students demonstrated a higher correct response rate (73%) than their engineering (53%) and social sciences (56%) counterparts. The youngest group of students had lower scores, averaging 7.54 points compared to the other three age categories which averaged around 8.62 points. While effect size estimates suggested small differences for sex and age, moderate effects were observed for the study program (Table 1).

**Figure 1:**
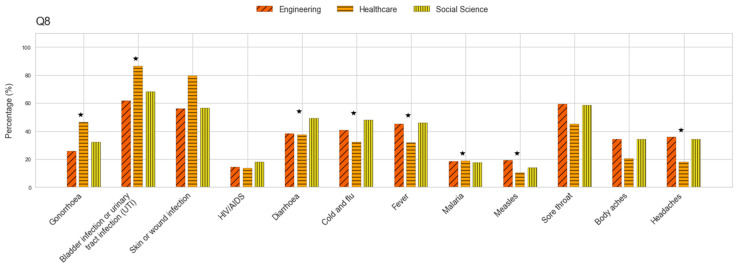
Percentage of respondents selecting each option for conditions presented as treated with antibiotics. ★ denotes significant differences.

The participants were largely aware that antibiotics should be taken as prescribed, as only 17% (n = 153) admitted to stopping their medication when they felt better (Q5). Three-quarters of the population (n = 695) disagreed with using antibiotics prescribed to a relative, even if intended for the same illness, while 14% (n = 126) agreed, and 11% (n = 101) were unsure (Q6). Similarly, when asked about using previously prescribed antibiotics for similar symptoms, 12% (n = 110) selected the “do not know” option, 62% (n = 576) recognized this as incorrect, and 25% (n = 236) agreed with the practice (Q7) (File S2, Supplementary Results, Data). Almost three-quarters of the participants (72%, n = 664) correctly associated UTIs with antibiotic use, while 62% (n = 575) did so for skin and wound infections, and 35% (n = 323) for gonorrhea (Q8). In particular, the percentage of individuals selecting correct answers was higher among the healthcare students than among those in engineering and social studies (Figure 1). More than half of the students (55%, n = 508) incorrectly identified sore throat as a condition treatable with antibiotics, while 44% (n = 413) selected diarrhea, 42% (n = 389) chose fever, and 42% (n = 396) selected cold and flu. The latter option was more commonly chosen by the social science and engineering students compared to those associated with healthcare (Figure 1). Similarly, these students were more likely to incorrectly associate headaches with antibiotic use than those in healthcare. Interestingly, antibiotics were also mistakenly linked to other conditions, including malaria and AIDS (Figure 1) (File S2, Supplementary Results, Data).

Table 2 presents the percentages of correct and incorrect responses given by students to statements about antibiotics (Q11). Nearly three-quarters of the participants accurately acknowledged that infections are becoming more resistant, that treating resistant bacteria is increasingly difficult, and that such infections could impact both themselves and their relatives. They also recognized this issue as a national concern. In addition, 67% (n = 618) of the respondents understood that antibiotic resistance heightens the risks associated with medical procedures such as surgery, organ transplants, and cancer treatment. Conversely, only 13% (n = 125) correctly understood that antibiotic resistance does not result from the body becoming resistant to drugs, while nearly half (45%, n = 418) mistakenly associated the issue with individuals who frequently take antibiotics. Moreover, only 40% of the participants agreed that resistant bacteria can spread between people (n = 373) and that antibiotics are extensively used in agriculture in the country (n = 376) (File S2, Supplementary Results, Data).

### 2.5. Attitudes Regarding Concerns About the Use of Antibiotics

**Figure 2:**
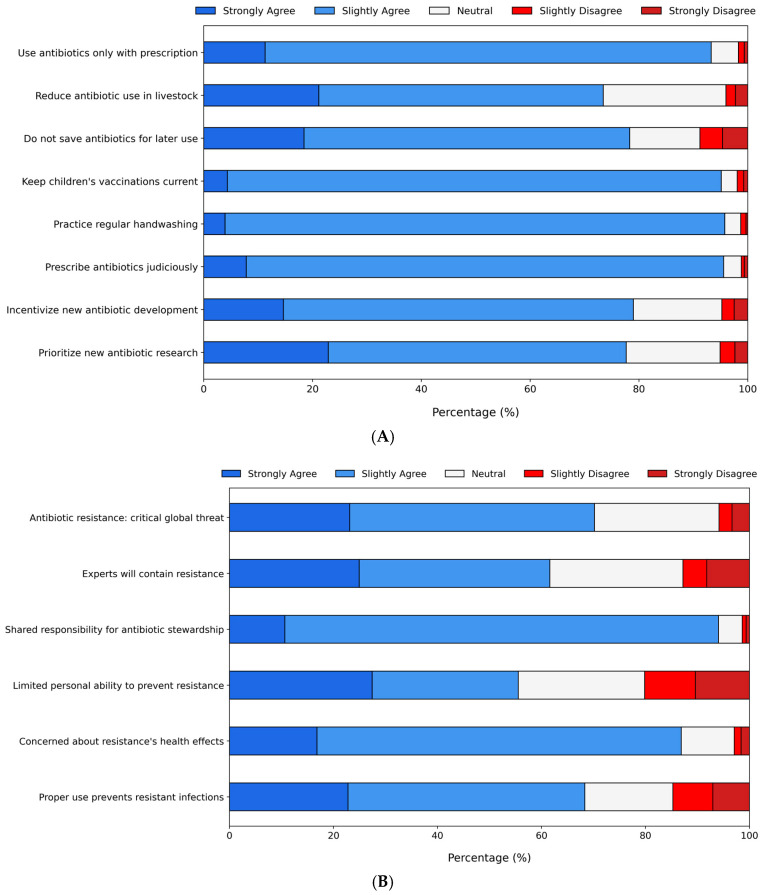
Likert scale results reflecting student perspectives on (**A**) potential actions to address the antibiotic resistance issue and (**B**) additional statements related to attitudes toward antibiotic resistance.

The participants were also presented with potential actions to address the antibiotic resistance issue (Q12) (Figure 2A). Around 22% were neutral or disagreed not only with the idea that people store antibiotics for later use but also with the need for government and pharmaceutical company involvement in developing new antibiotics. Moreover, 27% expressed neutrality or disagreement regarding the reduction in antibiotic use in food-producing animals. On the other hand, over 90% of the students agreed that antibiotics should only be used with a professional prescription and restricted to necessary cases. They also supported preventive measures such as regular handwashing and ensuring childhood vaccination by parents. Further statements were included to evaluate participants’ perspectives and attitudes (Q13) (Figure 2B). The majority (>90%) agreed that everyone shares responsibility for using antibiotics appropriately and expressed concern about the impact of resistant bacteria on their health and that of their families. Likewise, more than 90% of the students agreed that everyone must take responsibility for proper antibiotic use. While 70% recognized antibiotic resistance as a major societal issue, the same percentage believed they were not personally at risk if antibiotics were used correctly. Around 40% disagreed that medical experts alone would resolve the problem, and a similar proportion were neutral or disagreed with the notion that ordinary individuals have little influence in addressing the issue.

## 3. Discussion

This study investigated the use, sources of information, knowledge, and attitudes regarding AMR among a general population of students in two universities located in Samborondón, Greater Guayaquil. Most students reported obtaining antibiotics from professionals, receiving appropriate guidance, and primarily purchasing them from medical stores. The participants were familiar with common terminology related to antibiotic resistance and cited physicians and teachers as their main sources of information. The correct response rate was 56%, with the respondents often associating antibiotic use with conditions where they are not typically utilized. Overall, the students exhibited positive attitudes toward potential actions aimed at addressing the issue of antibiotic resistance.

The response rate was evaluated through both true-or-false and multiple-choice questions. Previous research classified knowledge levels based on the percentage of correct responses, defining them as acceptable (≥80%), moderate (60–80%), and low (<60%) [18]. In contrast, another study suggested a similar categorization but with different thresholds, defining acceptable, moderate, and low levels as ≥71%, 42–70%, and ≤42%, respectively [30]. According to these parameters, the observed level of knowledge can be considered low to moderate. Studies conducted among university students in various countries have also reported moderate levels of knowledge [18,21,30,33]. In Ecuador, a moderate correct response rate has been identified among both medical students and a broader university student population [25,27]. As demonstrated in previous studies [22,27,33,34,35], our findings indicate that males and healthcare students achieved higher scores, whereas younger students and those from social sciences and engineering scored lower. A similar trend has also been observed in Ecuadorian students regarding knowledge of COVID-19 and genetics [28,29]. A moderate AMR knowledge score among university students highlights gaps in their understanding that may contribute to the misuse of antibiotics in their future careers. This lack of awareness can undermine public health efforts, antibiotic stewardship, and policy decisions. Without proper knowledge, graduates risk engaging in improper prescribing and self-medication, worsening resistance.

Nearly 72% linked UTIs to antibiotic use, 62% did so for skin infections, and 35% for gonorrhea, with higher accuracy among healthcare students. Over half incorrectly identified sore throat, 44% chose diarrhea, and 42% selected fever or cold and flu, with the social science and engineering students making more errors. They also misassociated headaches with antibiotic use more often than the healthcare students. Surprisingly, antibiotics were wrongly linked to malaria and AIDS. These misconceptions reflect Ecuador’s historical over-the-counter antibiotic culture and educational gaps regarding infection management. Many students associate antibiotics with symptom relief (e.g., fever and headache), as previously documented in a previous study carried out in Quito [27]. Similar errors have been observed among undergraduate students from different backgrounds [22,27,33,35,36]. The misattribution of antibiotics to unrelated conditions may lead to inappropriate self-medication or, in certain professions, overprescription. These misunderstandings could influence Ecuador’s antimicrobial resistance problem by normalizing antibiotic misuse. Future health professionals may overprescribe due to ingrained beliefs, while non-clinical graduates could underprioritize antimicrobial resistance solutions. The cycle could persist across generations without intervention. We propose mandatory AMR education, following successful regional models, to break this pattern. Moreover, only 13% correctly identified that resistance is not caused by the body, and around 40% acknowledged bacterial transmission between people and widespread antibiotic use in agriculture. The limited awareness of agricultural antibiotic use among the students might stem from deficiencies in One Health education and minimal public discourse about the role of agriculture in antimicrobial resistance. This knowledge gap is particularly significant given Ecuador’s livestock production sector. Future professionals lacking this awareness may fail to advocate for or adhere to crucial interventions such as antibiotic use regulations in agriculture. Integrating One Health principles into university curricula could address this critical gap. These findings highlight the urgent need for targeted educational reforms to improve AMR literacy and promote responsible practices, especially in students not associated with medical sciences, as has been suggested previously [37,38,39]. Strengthening AMR-related training will help build a solid foundation in appropriate antibiotic use, ensuring that future professionals are well-equipped to address this issue.

Although the participants had a moderate level of knowledge, they still followed recommended practices. Over 80% of the students used antibiotics in the past year. The antibiotic use (37%) among university students may reflect Ecuador’s soft prescription enforcement and cultural tendencies toward self-medication or preventive use for viral illnesses. Moreover, limitations such as self-reporting bias (e.g., misclassified medications) or undetected chronic conditions (e.g., acne and UTIs) in our sample may also contribute to this particularity. While most obtained them from professionals, a quarter of them used other sources. General practitioners provided guidance in most cases, but 15% took antibiotics without advice. Pharmacies were the main source, though some relied on home storage or others. Correct practices, as well as incorrect ones, have been documented in similar studies in Ecuador [14,15,27] and other countries [32,40]. Various socioeconomic factors influence behavior regarding antibiotic use, especially in developing societies. Weak regulations, public demand, misinformation, and improper prescribing are among the main contributors to the problem [41,42,43]. Safe practices have been shown to improve with access to accurate information, not only in the context of antibiotic resistance but also for other major infectious diseases [16,44,45,46]. Nevertheless, studies have indicated that enhancing knowledge alone does not guarantee behavior correction or adherence to proper practices [47,48].

In general, terms such as antibiotic resistance, antibiotic-resistant bacteria, superbugs, AMR, and drug resistance were familiar to the majority of interviewees, who cited doctors and educators as their primary sources of information on these topics. Comparable results have been observed in university students, who identified physicians and educators as their primary sources of information [27,32]; this implies that introducing courses will enhance students’ understanding and engagement with essential concepts of responsible antibiotic use. Research has emphasized the effectiveness of educational campaigns in raising awareness and shaping behaviors [49,50]. Interestingly, specific campaigns were among the least chosen options as reported by a previous study among Ecuadorian students [27]. Consequently, it is recommended to create and launch educational campaigns on antibiotic use, delivering accurate health information through mainstream communication platforms, including social media.

The students mostly supported actions against antibiotic resistance, such as proper prescriptions and preventive measures. However, about a quarter were neutral or disagreed with reducing antibiotic use in farming, similar perspectives have been evidenced in students of different countries [27,32,40]. These perspectives could obstruct efforts to address AMR effectively and shape future policies. Educating students on these issues is vital for altering perceptions and fostering responsible antibiotic practices. This will be essential for safeguarding both public and animal health. Additional statements also show that most agreed that everyone is responsible for proper antibiotic use and expressed concern about resistance. While 70% saw it as a major issue, they felt personally safe if antibiotics were used correctly, which has been shown not to be the case [51]. We observed that nearly half of the students disagreed with the notion that individuals have little influence on the antibiotic issue. These misconceptions have been documented among other populations of university students [27,32]. The public is likely unaware of the impact they can have on this issue. Therefore, educational initiatives should highlight the necessity of collective efforts, bringing together citizens, policymakers, and professionals in health and agronomy to address the problem effectively.

Overall, the outcomes observed in this study have previously been reported in similar investigations conducted in various countries [27,33,35,36,52]. Hence, the recommended strategies, such as mandatory courses, preventive measures, and public health campaigns, are relevant to regions with similar economic and social contexts. The creation of a network responsible for monitoring and assessing local AMR trends in hospitals and districts has been suggested as a viable strategy [53,54]. Furthermore, there has been strong support for including AMR-focused courses within undergraduate curricula [37,55,56]. These recommendations highlight potential actions that could help address the lack of AMR awareness effectively.

Several limitations of this study should be noted. First, while the research aimed not only to develop an initial understanding of antibiotic resistance, its use, sources of information, knowledge, and attitudes among university students but also to generate quantitative data on the topic within Greater Guayaquil, the findings cannot be generalized to other regions or populations. This is particularly relevant given the variability in educational standards across the country. Second, due to the cross-sectional design of the study, no causal inferences can be derived from the results. Finally, for future research, the use of probability sampling is recommended, as the measured knowledge may be overestimated given that the study relied on voluntary responses.

## 4. Materials and Methods

### 4.1. Ethics Approval

All the procedures involving humans were carried out following the Declaration of Helsinki. The data collected did not put the participants at risk as it was voluntary and anonymous. The interviewees provided informed consent before participation (File S3, Supplementary Materials, Informed consent) and could leave the study at any time. The Human Research Ethics Committee of the University of Las Américas (CEISH-UDLA) reviewed the research protocol, which has been waived from evaluation by CEISH in accordance with the applicable legal regulations (Reference number: 2023-EXC-023).

### 4.2. Survey Questionnaire

The “Antibiotic Resistance, Multi-country Public Awareness Survey” [57] was designed to evaluate public awareness of antibiotic resistance and has been effectively utilized in multiple studies [27,30,58,59]. The questionnaire was reproduced with permission from the WHO (ID: 202402031) and presented in Spanish. It consisted of five parts: (1) demographics, (2) use, (3) knowledge, (4) sources of information, and (5) attitudes (File S4, Supplementary Materials, Survey). Sex (male, female, or other), age (≤18, 19–21, 22–24, or ≥25), and study program (healthcare, engineering, or social sciences) comprised the demographic data. Healthcare covered fields related to medicine, dentistry, or pharmacy, while engineering embraced all the engineering disciplines. In contrast, social sciences represented programs focused on studying individuals and societies, including disciplines such as politics, linguistics, law, communication, and history. Except for the demographic section, the questionnaire primarily used multiple-choice formats. The knowledge component also included true or false (T/F) items. Correct answers were awarded one point, while incorrect and “do not know” responses received no points, with a maximum possible score of 15. The survey was pre-tested (n = 20 students), and necessary adjustments were made before it was distributed to participants.

### 4.3. Study Setting, Participant Recruitment, and Procedure

This study took place from August to November 2024 among university students from two different institutions in Samborondón, Greater Guayaquil. Samborondón is a suburban district in Greater Guayaquil, Ecuador’s largest metropolitan area on the Pacific coast. The region blends urban and semi-rural characteristics, hosting universities that serve diverse socioeconomic groups. Two universities were selected as representative urban institutions in Samborondón with diverse student demographics suitable for our antibiotic awareness study, while also offering practical research access. To maintain ethical standards and minimize bias, the names of the universities in this study remain anonymous, guaranteeing confidentiality, generalizability, and institutional privacy. The sample size was estimated using the online calculator “Raosoft” [60], with a margin of error of 5%, a confidence level of 95%, and a response distribution of 50%. At the time of testing, one institution had a total student population of 17,158, while the other had 14,384. Thus, a sample of 376 students was required per university. The number of correctly completed forms collected was 531 from one institution and 391 from the other. The selection of participants was based on non-random criteria (participation was voluntary) since the study sought to gain preliminary insights into awareness of antibiotic resistance among university students from different study programs. The online survey, developed in Spanish on Google Forms, was distributed to the students through the official communication channels of each university in order to optimize data collection efficiency and participant accessibility. Potential self-selection bias, associated with online data collection, was mitigated through sending multiple reminders to non-respondents. The participants were given guidance on how to complete the questionnaire, and the correct answers were made available once the survey was submitted.

### 4.4. Statistical Analyses

Percentages and frequencies were used to describe the data. Levene’s test and Shapiro–Wilk’s test assessed the homogeneity of variance and normality, respectively. As data from all the demographics (sex, age, and program of study) showed equal variance but were non-normally distributed, the Kruskal–Wallis method was used. Medians were utilized to depict the center of distribution. Moreover, effect size measures, along with their confidence intervals, were assessed to gain information on the magnitude of differences between demographic groups. Values of 0.01, 0.06, and 0.14 for the calculated Eta Squared (η2) show small, medium, and large differences, respectively [61,62]. The chi-square test was employed to compare frequencies between population categories. Significance was set as *p* ≤ 0.05. The analyses were carried out in MATLAB^®^ version 9.9.9341360 (MathWorks, Natick, MA, USA), and the figures were produced using Python’s plotting library, Matplotlib 3.0.3 (Python Software Foundation, Fredericksburg, VA, USA).

## 5. Conclusions

The present findings show that the overall knowledge could be considered moderate. The healthcare students demonstrated significantly higher scores compared to their engineering and social sciences counterparts. Although many students demonstrated correct antibiotic practices, persistent misconceptions included using antibiotics for unrelated conditions, misunderstanding resistance as a patient trait rather than microbial adaptation, and unawareness of agricultural use. While most participants reported following the recommended practices and expressed positive attitudes toward addressing resistance, their primary information sources were healthcare professionals or teachers, and not educational campaigns. These findings identify crucial antibiotic knowledge gaps in university populations, guiding future AMR education initiatives. Subsequent studies should assess long-term knowledge retention through longitudinal tracking and evaluate targeted stewardship interventions, such as integrated One Health courses, across academic disciplines.

## Figures and Tables

**Table 1:** Demographics and scores.

Variables	Number of Participants	%	Median Score (Maximum 15)	F-Values	*p*-Value	η2—Eta Squared	95% CI **	
*Sex*				16.58	<0.001	0.02	0.004	0.04
Female	603	65.40	8.00 (4.1)					
Male	319	35.60	9.00 (3.8)					
*Age*				17.88	<0.001	0.02	0.004	0.04
≤18	136	14.75	7.54 (3.1)					
19–21	271	29.39	8.45 (4.4) *					
22–24	130	14.10	8.50 (4.1) *					
≥25	385	41.76	8.90 (3.9) *					
*Study Program*				90.93	<0.001	0.09	0.06	0.14
Healthcare	237	25.70	10.91 (4.1)					
Engineering	120	13.02	7.89 (4.5) *					
Social sciences	565	61.28	8.36 (4.3) *					

Medians are presented along with their interquartile range (IQR). * significant differences with the “≤18” group and with the “Healthcare” group. ** confidence intervals for η2.

**Table 2:** Statements used to assess antibiotic knowledge among undergraduates.

	Correct Answers	%	Incorrect Answers	%
Q11.1 Antibiotic resistance occurs when your body becomes resistant to antibiotics and they no longer work well (F)	125	13.56	797	86.44
Q11.2 Many infections are becoming increasingly resistant to treatment with antibiotics (T)	717	77.77	205	22.23
Q11.3. If bacteria are resistant to antibiotics, it can be very difficult or impossible to treat the infections they cause (T)	696	75.49	226	24.51
Q11.4. Antibiotic resistance is an issue that could affect me or my family (T)	690	74.84	232	25.16
Q11.5. Antibiotic resistance is an issue in other countries but not in Ecuador (F)	674	73.10	248	26.90
Q11.6. Antibiotic resistance is only a problem for people who take antibiotics regularly (F)	418	45.34	504	54.66
Q11.7 Bacteria resistant to antibiotics can be spread from person to person (T)	373	40.46	549	59.54
Q11.8. Antibiotic-resistant infections could make medical procedures like surgery, organ transplants, and cancer treatment more dangerous (T)	618	67.03	304	32.97
Q11.9. In Ecuador, antibiotics are widely used in agriculture and farms (T)	376	40.78	546	59.22

Correct options are indicated in parentheses.

## Data Availability

The data presented in this study are available upon request from the corresponding author.

## Supplementary Materials

The following supporting information can be downloaded at https://www.mdpi.com/article/10.3390/antibiotics14050440/s1, File S1: Supplementary Results, Answers; File S2: Supplementary Results, Data; File S3: Supplementary Materials, Informed consent; File S4: Supplementary Materials, Survey.
