# Supplementary material for: A Cross-Sectional Study Assessing Antibiotic Resistance Awareness Among University Students in Samborondón, Greater Guayaquil, Ecuador
Source: Antibiotics (Basel). 2025 Apr 27;14(5):440. doi: 10.3390/antibiotics14050440 (PMC12108333; doi:10.3390/antibiotics14050440)
Supplement: Supplementary file 1 [file antibiotics-14-00440-s001.zip › Supplementary Materials, Informed consent, File S3.pdf]

Informed Consent for Anonymous Surveys

INTRODUCTION AND PROCEDURE DESCRIPTION:

This research is led by Dr. Marco Larrea-Álvarez, PhD, professor/researcher at UEES and principal investigator of the study *"Knowledge, attitudes, and practices regarding antibiotic resistance, alternatives, and the role of the genome in its metabolism among students from two universities in the city of Guayaquil."* This study is part of the project *"Prevalence of antibiotic-resistant E. coli in key areas such as the environment, community, and hospital in the Ecuadorian coastal region, and the relationship of resistance to mobile genetic elements."* The study also has the assistance of Dr. Miroslava Anna Sefcova, PhD.

PURPOSE OF THE STUDY: To determine the level of understanding of antibiotic resistance, its potential alternatives, and the role of genomics in its metabolism among students from two universities in the city of Guayaquil.

PARTICIPANTS: Undergraduate and postgraduate students from two universities in the city of Guayaquil.

RISKS AND BENEFITS: There are no risks associated with this survey. All information will remain anonymous. There are no direct benefits for participating in this study. However, the information provided may help healthcare professionals better understand the level of comprehension of antibiotic resistance in a key population.

ANONYMITY: Your participation in this research is completely anonymous. None of the information you share can be traced back to you. Data will be securely stored in the database of the online survey platform. Only the principal investigator and the technical assistant will have access to the information.

VOLUNTARY PARTICIPATION IN THE STUDY: Your participation is voluntary, and you may choose not to participate or withdraw at any time.

COSTS AND COMPENSATION: There is no compensation for participating in this study.

CONTACT INFORMATION: You may contact the researchers involved at any time. For this purpose, you can email them at: marcolarrea@uees.edu.ec y miroslava.sefcova@gmail.com.

USE OF INFORMATION: The researchers will use the responses obtained in this study for scientific research purposes. The study is anonymous. The collected information will be used exclusively for the proposed scientific research and, only when explicitly approved, for future related studies. Any further research will require a new informed consent form approved by a Research Ethics Committee recognized by the Ministry of Public Health.

If you wish to participate in this study, click the **"Next"** button (bottom left) to begin the survey and answer the attached questions. By clicking the **"Next"** button, you consent to participate.

Thank you,

***Marco Andrés Larrea-Álvarez, PhD*.**
Principal Investigator
