# Supplementary material for: A Cross-Sectional Study Assessing Antibiotic Resistance Awareness Among University Students in Samborondón, Greater Guayaquil, Ecuador
Source: Antibiotics (Basel). 2025 Apr 27;14(5):440. doi: 10.3390/antibiotics14050440 (PMC12108333; doi:10.3390/antibiotics14050440)
Supplement: Supplementary file 1 [file antibiotics-14-00440-s001.zip › Supplementary Materials, Survey, File S4.pdf]

A cross-sectional study: Antibiotic resistance awareness among university students in Greater Guayaquil, Ecuador

Section 1: Demographic data

Please mark with an X the appropriate answer:

I. What is your age?

☐ 18 or younger
☐ 19-21
☐ 22-24
☐ 25 or older

II. What is your gender?

☐ Male
☐ Female
☐ Other:

III. Program?

☐ Applied science
☐ Social science

Section 2: USE OF ANTIBIOTICS

Q1. When did you last take antibiotics?

- ☐ In the last month
- ☐ In the last 6 months
- ☐ In the last year
- ☐ More than a year ago
- ☐ Never
- ☐ Can't remember
- *If 'Never', go straight to Question 5*

Q2. On that occasion, did you get the antibiotics (or a prescription for them) from a doctor or nurse?

- ☐ Yes
- ☐ No
- ☐ Can't remember

Q3. On that occasion, did you get advice from a doctor, nurse or pharmacist on how to take them?

- ☐ Yes, received advice on how to take them (e.g. with food, for 7 days)
- ☐ No
- ☐ Can't remember

Q4. On that occasion, where did you get the antibiotics?

- ☐ Medical store or pharmacy
- ☐ Stall or hawker
- ☐ The internet
- ☐ Friend or family member
- ☐ I had them saved up from a previous time
- ☐ Somewhere/someone else
- ☐ Can't remember

Section 3: KNOWLEDGE ABOUT ANTIBIOTICS (part I.)

Q5. When do you think, you should stop taking antibiotics once you’ve begun treatment?

- ☐ When you feel better
- ☐ When you’ve taken all of the antibiotics as directed
- ☐ Don’t know

Q6. Do you think this statement is ‘true’ or ‘false’? “It’s okay to use antibiotics that were given to a friend or family member, as long as they were used to treat the same illness”

- ☐ True
- ☐ False
- ☐ Don’t know

Q7. Do you think this statement is ‘true’ or ‘false’? “It’s okay to buy the same antibiotics, or request these from a doctor, if you’re sick and they helped you get better when you had the same symptoms before”

- ☐ True
- ☐ False
- ☐ Don’t know

Q8. Do you think these conditions can be treated with antibiotics?

- ☐ HIV/AIDS
- ☐ Gonorrhoea
- ☐ Bladder infection or urinary tract infection (UTI)
- ☐ Diarrhoea
- ☐ Cold and flu
- ☐ Fever
- ☐ Malaria
- ☐ Measles
- ☐ Skin or wound infection
- ☐ Sore throat
- ☐ Body aches
- ☐ Headaches

Section 4: SOURCE OF INFORMATION

Q9. Have you heard of any of the following terms

☐ Antibiotic resistance
☐ Superbugs
☐ Antimicrobial resistance
☐ AMR
☐ Drug resistance
☐ Antibiotic-resistant bacteria
☐ I have not heard

10.1. Ask if answered YES @ 9) to 'Antibiotic Resistance' Where did you hear about the term: **'Antibiotic Resistance'?**

☐ Doctor or nurse
☐ Teacher or researcher
☐ Pharmacist
☐ Family member or friend (including on social media)
☐ Media (newspaper, TV, radio)
☐ Specific campaign
☐ Other
☐ Can't remember

10.2. Ask if answered YES @ 9) to 'Superbugs' Where did you hear about the term: **'Superbugs'?**

☐ Doctor or nurse
☐ Teacher or researcher
☐ Pharmacist
☐ Family member or friend (including on social media)
☐ Media (newspaper, TV, radio)
☐ Specific campaign
☐ Other
☐ Can't remember

10.3. Ask if answered YES @ 9) to 'Antimicrobial Resistance' Where did you hear about the term: **'Antimicrobial resistance'?**

☐ Doctor or nurse
☐ Teacher or researcher

☐ Pharmacist
☐ Family member or friend (including on social media)
☐ Media (newspaper, TV, radio)
☐ Specific campaign
☐ Other
☐ Can't remember

10.4. Ask if answered YES @ 9) to 'AMR' Where did you hear about the term: **'AMR'?**

☐ Doctor or nurse
☐ Teacher or researcher
☐ Pharmacist
☐ Family member or friend (including on social media)
☐ Media (newspaper, TV, radio)
☐ Specific campaign
☐ Other
☐ Can't remember

10.5. Ask if answered YES @ 9) to 'Drug resistance' Where did you hear about the term: **'Drug resistance'?**

☐ Doctor or nurse
☐ Teacher or researcher
☐ Pharmacist
☐ Family member or friend (including on social media)
☐ Media (newspaper, TV, radio)
☐ Specific campaign
☐ Other
☐ Can't remember

10.6. Ask if answered YES @ 9) to 'Antibiotic-resistant bacteria' Where did you hear about the term: **'Antibiotic-resistant bacteria'?**

☐ Doctor or nurse
☐ Teacher or researcher
☐ Pharmacist
☐ Family member or friend (including on social media)
☐ Media (newspaper, TV, radio)
☐ Specific campaign
☐ Other
☐ Can't remember

Section 3: KNOWLEDGE ABOUT ANTIBIOTICS (part II.)

Q.	**Please indicate whether you think the following statements are 'true' or 'false'**	True	False	Don't know
11.1	Antibiotic resistance occurs when your body becomes resistant to antibiotics and they no longer work as well			
11.2	Many infections are becoming increasingly resistant to treatment by antibiotics			
11.3	If bacteria are resistant to antibiotics, it can be very difficult or impossible to treat the infections they cause			
11.4	Antibiotic resistance is an issue that could affect me or my family			
11.5	Antibiotic resistance is an issue in other countries but not here			
11.6	Antibiotic resistance is only a problem for people who take antibiotics regularly			
11.7	Bacteria which are resistant to antibiotics can be spread from person to person			
11.8	Antibiotic-resistant infections could make medical procedures like surgery, organ transplants and cancer treatment much more dangerous			
11.9	In Ecuador, antibiotics are widely used in agriculture (including food-producing animals)			

Section 5: ATTITUDES

Q.	**On the scale shown, how much do you agree the following actions would help address the problem of antibiotic resistance?**	**Agree Strongly**	**Agree Slightly**	**Neither agree nor disagree**	**Disagree Slightly**	**Disagree Strongly**
12.1	People should use antibiotics only when they are prescribed by a doctor or nurse					
12.2	Farmers should give fewer antibiotics to food-producing animals					
12.3	People should not keep antibiotics and use them later for other illnesses					
12.4	Parents should make sure all of their children's vaccinations are up-to-date					
12.5	People should wash their hands regularly					
12.6	Doctors should only prescribe antibiotics when they are needed					
12.7	Governments should reward the development of new antibiotics					
12.8	Pharmaceutical companies should develop new antibiotics					

Q.	**On the scale shown, how much do you agree with following statements?**	**Agree Strongly**	**Agree Slightly**	**Neither agree nor disagree**	**Disagree Slightly**	**Disagree Strongly**
13.1	Antibiotic resistance is one of the biggest problems the world faces					
13.2	Medical experts will solve the problem of antibiotic resistance before it becomes too serious					
13.3	Everyone needs to take responsibility for using antibiotics responsibly					
13.4	There is not much people like me can do to stop antibiotic resistance					
13.5	I am worried about the impact that antibiotic resistance will have on my health, and that of my family					
13.6	I am not at risk of getting an antibiotic-resistant infection, as long as I take my antibiotics correctly.					
